# Greedy Data Transportation Scheme with Hard Packet Deadlines for Wireless Ad Hoc Networks

**DOI:** 10.1155/2014/815123

**Published:** 2014-09-01

**Authors:** HyungJune Lee

**Affiliations:** Department of Computer Science and Engineering, Ewha Womans University, Seoul 120-750, Republic of Korea

## Abstract

We present a greedy data transportation scheme with hard packet deadlines in ad hoc sensor networks of stationary nodes and multiple mobile nodes with scheduled trajectory path and arrival time. In the proposed routing strategy, each stationary ad hoc node en route decides whether to relay a shortest-path stationary node toward destination or a passing-by mobile node that will carry closer to destination. We aim to utilize mobile nodes to minimize the total routing cost as far as the selected route can satisfy the end-to-end packet deadline. We evaluate our proposed routing algorithm in terms of routing cost, packet delivery ratio, packet delivery time, and usability of mobile nodes based on network level simulations. Simulation results show that our proposed algorithm fully exploits the remaining time till packet deadline to turn into networking benefits of reducing the overall routing cost and improving packet delivery performance. Also, we demonstrate that the routing scheme guarantees packet delivery with hard deadlines, contributing to QoS improvement in various network services.

## 1. Introduction

Wireless ad hoc networks are considered as one of the promising future networks that do not require the existing infrastructure or centralized administration. As can be seen in recent technology trends such as M2M (machine-to-machine) and D2D (device-to-device), network devices are interconnected to each other and form a noncentralized network, called* ad hoc* network that allows direct data transmission over multihop relays.

These ad hoc networks consist of not only stationary nodes that establish a relatively stable multihop network, but also mobile nodes that increase the coverage of network (as message ferries [1]) and improve network capacity with a sacrifice of packet delay (proved in information theory [2]).

There are different types of data depending on applications. If the ad hoc networks deliver real time data from a source to a destination, the data needs to be taken with the highest priority to minimize network delay as much as they can. In case of nonreal time data delivery, on the other hand, the network can find many different ways to route data as long as it can meet the given packet deadline. Timely data delivery within a given packet deadline is an important requirement to guarantee QoS in various network services.

As energy efficiency becomes more important in the recent ubiquitous networks, finding more efficient ad hoc routes with less energy consumption has received significant attentions in research and industry [3–5]. In this context, we may consider some other detouring routes that incurs higher energy efficiency, that is, lower routing cost, even if the selected routes come with a sacrifice of packet delay increase within the given packet deadline.

In this paper, we study the problem of energy-efficient data transportation with packet deadlines in the ad hoc networks of stationary nodes and mobile nodes. We consider* multiple* mobile nodes serving as message ferries that move predefined paths with regular time schedules (e.g., city buses with regular interarrival times that have installed wireless network cards).

We propose a distributed greedy data transportation scheme in which involved intermediate ad hoc nodes en route decide whether to relay a next-hop stationary node toward destination or to a mobile node that will carry closer to destination. We aim to utilize mobile nodes to minimize the total routing cost for packet delivery throughout the multihop paths as far as the selected route can satisfy the end-to-end packet deadline.

Prior works on data delivery using mobile nodes as message ferries can be classified into two categories: (1) trajectory control of a single mobile relay for energy-efficient data collection (in [1, 6–8]) and (2) opportunistic routing in delay-tolerant networks (DTNs) (in [9–13]). More recently, research on time-sensitive opportunistic routing in DTN has been initiated in [14, 15]. However, the data delivery problem with* hard deadlines* in two-tier ad hoc networks with both stationary nodes and* multiple* mobile nodes (exploiting as relays) following predefined paths has been neither explicitly defined nor studied well yet.

We formally define the* data transportation* problem in stationary ad hoc networks with mobile nodes that follow predefined paths under the given deadline constraint. Simulation results show that our proposed routing algorithm fully exploits the remaining time till packet deadline to turn into networking benefits of reducing the overall routing cost and improving packet delivery performance, through selective utilization of mobile nodes as message ferries. Also, we demonstrate that the routing scheme guarantees packet delivery with hard deadlines.

Our main contributions can be summarized as follows.

- We introduce the problem of data transportation with hard deadlines in ad hoc networks of stationary nodes and mobile nodes.
- We propose a greedy routing strategy that reduces energy consumption for multihop packet transmission by selectively exploiting mobile nodes that can carry the packet closer to the destination given the deadline constraint.
- We show a tradeoff between energy consumption and packet deadline and provide an explicit way to turn available packet time into minimizing routing cost throughout the ad hoc network.

The rest of this paper is organized as follows: after we present system model in Section 2, our proposed data transportation scheme and routing protocol are presented in Section 3. In Section 4, we evaluate network performance of our algorithm compared to the shortest-path routing algorithm using only stationary networks, and finally this paper is concluded in Section 5.

## 2. System Model

This paper considers the problem of data delivery from a stationary source node to a stationary sink node in ad hoc networks. Stationary ad hoc nodes are deployed throughout the networks, and mobile nodes can connect to the stationary ad hoc networks while moving along their own predetermined moving paths. Either stationary or mobile node can transmit data to either stationary or mobile node if they are located within communication range. When a mobile node receives data from either a stationary node or another mobile node, it can carry the received data until it decides to relay them to a nearby node within its communication range.

The main objective of this work is to minimize packet transmission cost incurred by data delivery from source to destination by taking advantage of mobile nodes (i.e., message ferries) as long as the packet delay from the entire route is less than the given packet deadline. We use the stationary networks as the underlying lower level network, and mobile nodes make contacts with a part of the stationary nodes along their moving paths. The expected number of transmissions on a link is used as the link cost for the hop, and the cost for a route by Dijkstra's algorithm is the sum of the per-hop costs. It should be noted that other more efficient routing algorithms [16–18] can be used as the underlying stationary routing method.

The problem of data delivery that we aim to solve in this paper can then be described as finding a series of next relay nodes and their types between* stationary* and* mobile* (relaying from the previous stationary nodes), and also next drop-off stationary nodes (relaying from the previous mobile nodes).

## 3. Data Transportation Scheme

Ubiquitous network deployments allow the formation of a large scale stationary ad hoc network where data packet can be traversed with multihop transmission without any central network controllers. On top of these stationary ad hoc networks, there are different types of mobile nodes that can communicate with the stationary ad hoc networks as well as with other mobile nodes: (1) mobile nodes for the predefined regular paths such as public transport system of city bus and metro and (2) mobile nodes along nonregular paths. In this paper, we focus on the usage of the first type mobile nodes by extending the problem of message ferrying to multiple mobile relaying under packet deadlines.

**Figure 1:**
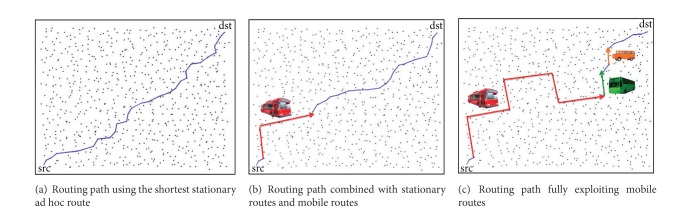
Routing paths from stationary source (in the left bottom) to stationary destination (in the right top) in the usages of stationary and mobile paths. Routing cost and packet deadline constraint determine which routing path should be selected for effective data transportation.

By exploiting regular mobile nodes as* data* transportation system, data packets would rather be carried on mobile nodes that will move closer to the destination without incurring additional transmission cost, as long as the selected route can meet a given packet deadline. For example, as shown in Figure 1, in case of data with a tight packet deadline, packets have no choice but to be delivered through stationary ad hoc networks while consuming significant multihop routing cost until the last delivery to destination. In case of data with somewhat relaxed packet deadline, on the other hand, if an intermediate stationary node en route can load data into a passing-by mobile node that will move toward destination, it would save routing cost while moving on the ferry. If the mobile node starts moving away from the destination after some point, it would rather* get off* the carried data packets at a nearby stationary node (i.e., relay the carried data packets from the mobile node to a stationary node within radio range). The stationary node continues to find an energy-efficient route given the remaining time until the deadline.

This mechanism is analogous to people's cost-effective transit decision in public transportation systems. For example, if a person is in a hurry, he will take a taxi along the shortest path toward destination even though it costs the very expensive fare; however, if a person has enough time to get to the destination, he may want to detour by taking buses even with longer routes, but incurring cheaper fare in total, as also analytically studied in transportation engineering [19].

In this section, we describe the proposed routing scheme by which an intermediate ad hoc node can determine an energy-efficient route toward destination by selecting the shortest next hop on stationary networks or loading into a* soon-passing-by* mobile node, given a packet deadline. As far as the entire route can meet the packet deadline, our proposed scheme encourages more mobile nodes to be involved with routing packets toward destination, leading to tremendous reduction in packet transmission cost.

### 3.1. Overview

We use the concept of* trajectory* of mobile nodes defined in [20]. A mobile node passes through stationary ad hoc networks on a given spatial path while sending periodic beacons and listening for replies. As the mobile node is being associated with a series of stationary nodes, the mobile node can record the associated node ID at each beacon time as a trajectory (sequence). For example, from the recorded trajectory of mobile node *i*,

(1) $$\begin{array}{c} T_{M_{i}}=N_{2}N_{2}N_{5}N_{5}N_{3}N_{3}N_{3}N_{3}N_{6},\ldots , \end{array}$$

we can know of* which* stationary node the mobile node is connected to at each beacon period.

Every stationary node has information of (1) passing-by mobile node list, (2) the corresponding future trajectory, and (3) arrival times of each mobile node. This implies that a stationary node can transmit data directly to a specific mobile node at the time the mobile node passes by if the stationary node decides to load data into the mobile node.

Regarding stationary network topology, each stationary node is assumed to have link information for all the stationary nodes in networks. Thus, it can find the shortest next hop toward a specific destination node through Dijkstra's algorithm.

Given connection information of mobile nodes and stationary ad hoc nodes, a stationary ad hoc node en route makes a decision of relaying to either a neighboring stationary ad hoc node via the corresponding stationary link or a mobile node via a mobile link that will be established if the mobile node comes to itself within radio range. For this decision, the stationary ad hoc node takes into account the estimated transmission cost up to the destination, which will be incurred by the respective selection, and selects the route that incurs the lowest estimated transmission cost.

When we calculate the estimated transmission cost for the delivery to a mobile node, we list up possible future drop-off cases at each specific future stationary ad hoc node and compute the estimated transmission cost for routing over the remaining hops. Then, we choose the mobile node and its drop-off stationary node with the lowest estimated transmission cost. If a next stationary node receives data from either a neighboring stationary node or the drop-off from a mobile node, it follows the same procedure above until the data reaches the destination.

### 3.2. Algorithm

Assuming that *n* mobile nodes *M*_1_, *M*_2_,…, *M*_*n*_ regularly pass by a stationary ad hoc node, the stationary node is given the information of trajectories of mobile nodes, *T*_*M*_1__, *T*_*M*_2__,…, *T*_*M*_*n*__. Let us define the arrival times of mobile node *i* at a stationary ad hoc node as *A*_*M*_*i*__^(1)^, *A*_*M*_*i*__^(2)^,…, *A*_*M*_*i*__^(*m*_*i*_)^ over one day where *A*_*M*_*i*__^(1)^ is the first scheduled arrival time and *A*_*M*_*i*__^(*m*_*i*_)^ is the last scheduled arrival time of the day, respectively, at the stationary node that the mobile node *i* visits.

We denote BI by the beacon interval and *D*_hop_ by per-hop delivery time (assuming constant packet size and fixed power allocation). *C*(*N*_*i*_, *N*_*j*_) denotes the routing cost from node *N*_*i*_ to node *N*_*j*_ in stationary ad hoc networks. *C*_*s*↔*m*_ denotes the routing cost from a mobile node to the best connectable stationary node and vice versa.

The initial packet transmission time at source toward destination is denoted by *t*_src_, and the last packet reception time at destination is denoted by *t*_dst_. If a data packet needs to satisfy a packet deadline of *τ*_due_, the condition of *t*_dst_ − *t*_src_ ≤ *τ*_due_ should hold. The header in data includes the field of the remaining time till deadline and is updated on each hop-by-hop delivery, considering per-hop delivery time *D*_hop_ and moving time while on ferry, given a selected path.

First, the shortest stationary route to destination is the default path and keeps the shortest path cost from the stationary node itself to destination. The next hop to deliver is given by Dijkstra's algorithm. And then, the stationary node finds possible paths using mobile nodes (i.e., relaying to a passing-by mobile node and dropping off at a stationary node) that lead to the lower routing cost than the shortest stationary route cost and are able to meet the packet deadline. If it turns out that there is no available mobile path, the stationary node decides to relay to the selected stationary node. Otherwise, the stationary node decides to relay to a mobile node with which the estimated routing cost is the minimum among other alternatives.

In case of relaying to a mobile node *i*, we need to calculate the waiting time until the next scheduled mobile node arrives. Assuming that *t*_*receivedAtNode*_ is the reception time when the intermediate stationary node receives data, the waiting time is given by *A*_*M*_*i*__^(1)^ − *t*_*receivedAtNode*_ if *t*_*receivedAtNode*_ ≤ *A*_*M*_*i*__^(1)^ or *A*_*M*_*i*__^(*j*)^ − *t*_*receivedAtNode*_ if *A*_*M*_*i*__^(*j*−1)^ < *t*_*receivedAtNode*_ ≤ *A*_*M*_*i*__^(*j*)^. Upon the arrival of the selected mobile node at the stationary node, the stationary node relays data to the mobile node while taking the time of *D*_hop_ and consuming the routing cost of *C*_*s*→*m*_. The mobile node carries data until it reaches the selected drop-off node, while being connected to a series of trajectory nodes where the traversal time between two consecutive nodes is the beacon interval and consuming no routing cost. Lastly, when the mobile node drops off at the selected drop-off node and relays to it, it takes the time of *D*_hop_. Refer to the proposed algorithm for more details in Algorithm 1.

As a result of Algorithm 1, if the dropOff node ends up with null, the stationary node relays data to the selected stationary nextHop node. Otherwise, the stationary node relays data to the selected mobile node (=nextHop) and drops off the data at the selected dropOff node that the mobile node will reach and get connected to. Once the next stationary node receives data either from the previous hop node or from the mobile node, it follows the same procedures above by running Algorithm 1.

The computation complexity of our proposed greedy routing algorithm is *O*(*NL*) where *N* is the number of mobile nodes passing by the stationary node and *L* is the trajectory length of mobile nodes.

## 4. Simulation Results

**Figure 2:**
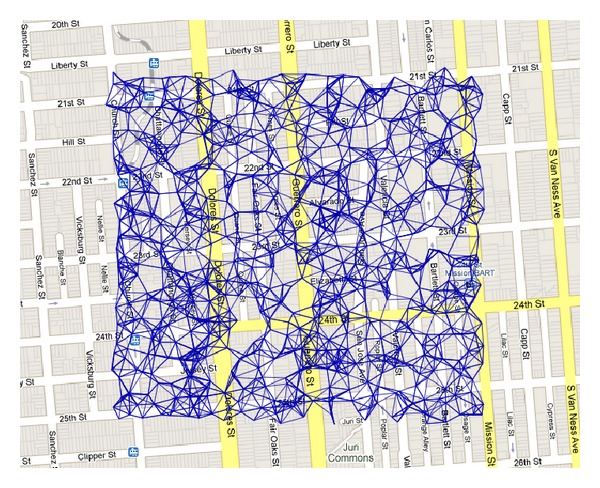
Connectivity graph over 716 sensor nodes where links are shown for PRR ≥ 75%. The average radio range is 36.2 m.

**Figure 3:**
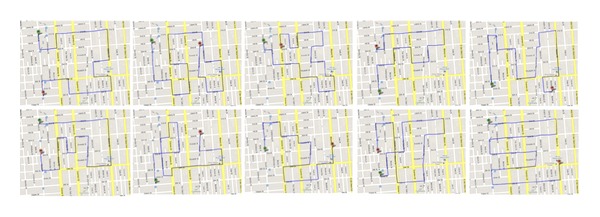
Moving paths of mobile vehicles (including the opposite direction as well). A total of 20 vehicles move over the network (as in Figure 2) at a constant speed of 30 km/h with regular time schedules while getting connected to stationary ad hoc nodes.

We evaluate the proposed greedy data transportation algorithm in sensor networks using simulated mobility data traces [20] where 716 stationary nodes are distributed over 830 × 790 m^2^ in a virtual downtown San Francisco (Figure 2) and 20 mobile nodes regularly move along the predefined paths (Figure 3). During their movements, they get connected to a part of stationary nodes within communication range. Each mobile node moves at a speed of 30 km/h and broadcasts beacons at every 1 second. To derive radio signal strengths at the receiving node from a transmitting node, a combined path-loss and shadowing model as the radio propagation model is used with a path-loss exponent of 3, a reference loss of 46.67 dB, and an additive white Gaussian noise of *N*(0, 5^2^) in dB [21]. Each mobile node sends beacons at every 1 second, receives responses from nearby stationary nodes, and records the node with the highest signal strength as a trajectory node. We use *D*_hop_ = 100 ms (assuming constant packet size of 25 kB and 250 kbps data rate based on 802.15.4) and *C*_*s*↔*m*_ = 1 (because data are delivered from mobile nodes to their best connectable stationary nodes and vice versa).

We validate our proposed algorithm using a MATLAB-based simulator that we implemented for the purpose of algorithmic validation. In this simulator, we do not explicitly simulate the MAC layer but rather focus on measuring the network layer performance. We evaluate network performance in terms of routing cost, packet delivery ratio, packet delivery time, and usability of mobile nodes. We examine how performance metrics vary with respect to different packet deadline constraints and mobile nodes' interarrival time. Data traffic is delivered from source to destination where they are far away from each other with the longest hop distance (as illustrated in Figure 1).

**Figure 4:**
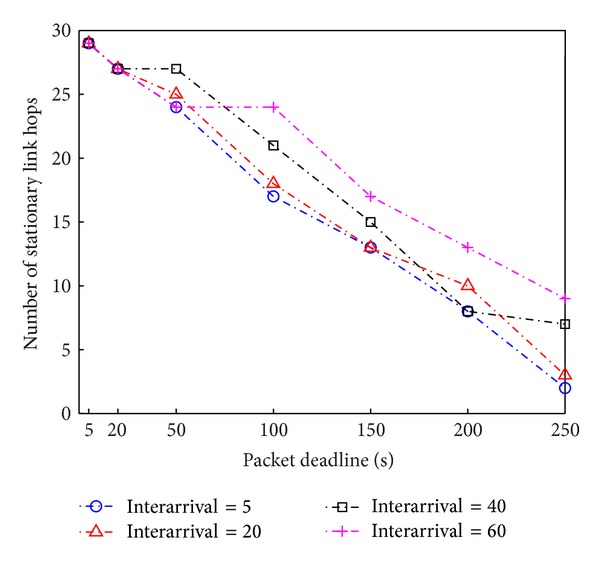
The number of stationary link hops with respect to packet deadline and mobile nodes' interarrival time.

First, we look into how the number of stationary link hops changes depending on the given packet deadline. For the tightest packet deadline case (i.e., a packet deadline of 5 seconds), the selected route does not utilize any mobile nodes for packet delivery and uses only stationary nodes for relaying. As shown in Figure 4, the packet traverses over 29 stationary-node hops from source to destination for a packet deadline of 5 seconds. As packets have more relaxed deadline constraints, our proposed algorithm prefers mobile ferries to stationary ad hoc nodes for packet relaying as long as the overall selected path results in a packet delay less than the packet deadline. Also, as the interarrival time of mobile nodes increases, the number of stationary link hops increases because the increased interarrival time increases the waiting time until the next scheduled mobile node arrives, and thus the chance of relaying to mobile nodes will be lowered to meet the packet deadline.

**Figure 5:**
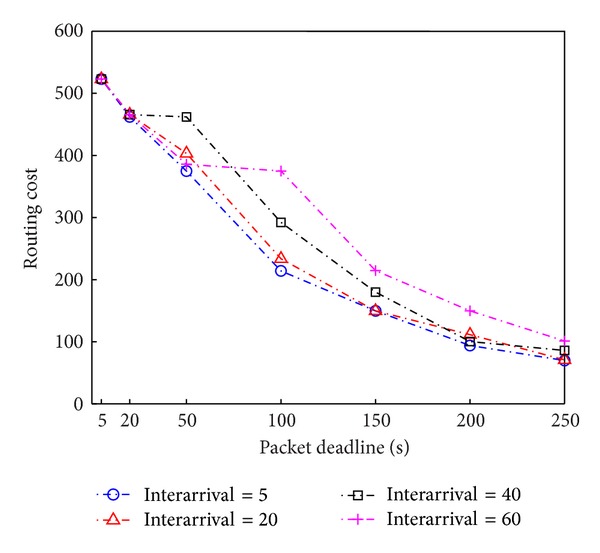
Routing cost with respect to packet deadline and mobile nodes' interarrival time.

We evaluate routing cost with respect to packet deadline and interarrival time of mobile nodes as in Figure 5. As packet deadline increases, our proposed algorithm tries to select mobile nodes for carrying data toward destination for the longer time in packet delivery, instead of traversing over many stationary nodes that consume much routing cost. Our proposed algorithm provides an explicit routing strategy for reducing significant amount of routing cost by selectively exploiting useful mobile ferries in the networks. As the interarrival time of mobile nodes decreases, we have more chance to use mobile nodes as the next relay node to carry data packet and drop off at a node closer to destination while consuming less routing cost.

**Figure 6:**
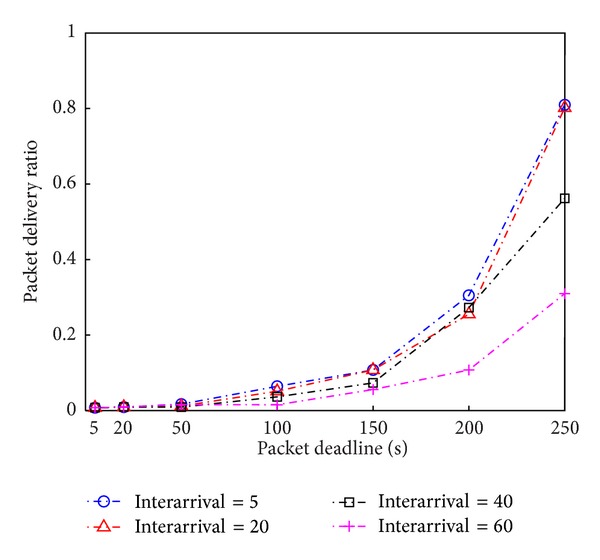
Packet delivery ratio with respect to packet deadline and mobile nodes' interarrival time.

We examine how our proposed algorithm can be helpful for improving packet delivery in a large scale network. If we use only stationary nodes as intermediate relay nodes to deliver data from source to destination where they are far away with 29 hops (for a packet deadline of 5 seconds), the resulting packet delivery ratio is very low because there are very high chances for data to be lost during 29-hop relaying (see Figure 6). As a longer packet deadline is allowed, our routing algorithm uses longer mobile paths, leading to reducing the number of stationary hop links and, thus, greatly improving packet delivery. For example, if the packet deadline of 250 seconds is permitted, the proposed routing algorithm achieves high packet delivery above 80% without any retransmissions, as opposed to the packet delivery of less than 1% for the packet deadline of 5 seconds. As the interarrival time of mobile nodes decreases, a higher chance of using mobile nodes achieves a more reliable packet delivery.

**Figure 7:**
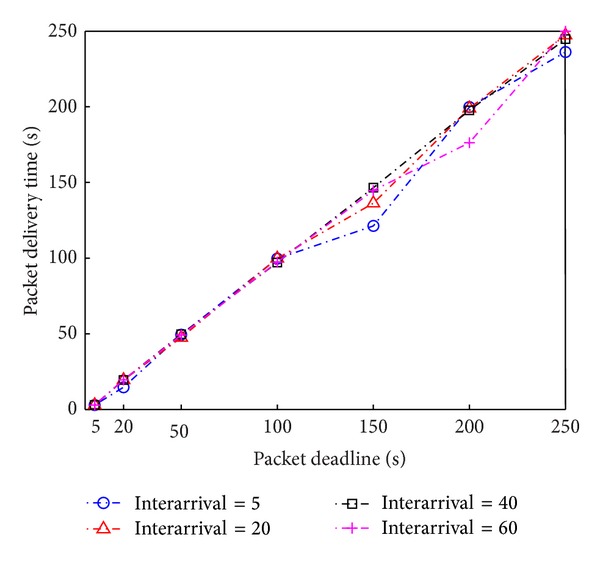
Packet delivery delay with respect to packet deadline and mobile nodes' interarrival time.

We investigate whether our routing algorithm guarantees packet delivery within a given packet deadline.  Figure 7 shows that the incurred packet delay while traversing over the selected path is less than the given packet deadline in all cases. This means that our algorithm can successfully deliver data from source to destination while satisfying hard packet deadlines. One more interesting property is that the incurred packet delay is very slightly less than the packet deadline, demonstrating that our routing algorithm maximizes the utilization of mobile nodes to minimize routing cost. Our routing method provides an explicit way to best utilize mobile nodes for delay-constrained packet routing.

**Figure 8:**
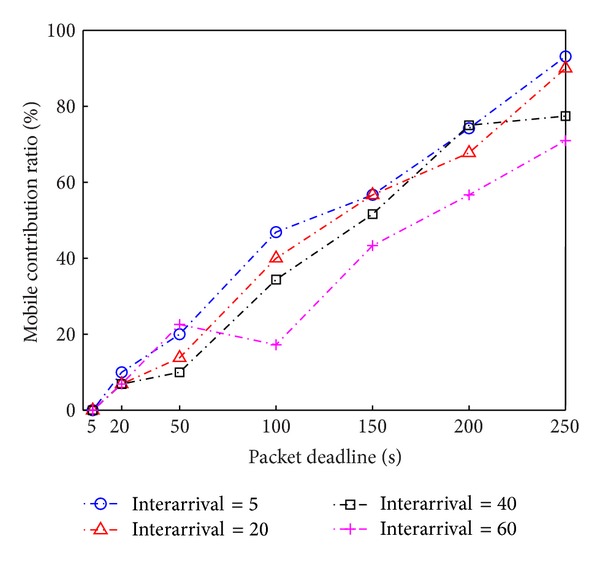
Contribution ratio for routing with mobile nodes with respect to packet deadline and mobile nodes' interarrival time.

We measure the contribution percentage of mobile nodes involved with the overall routing. We calculate the number of corresponding stationary hops for the selected mobile paths. This means that using the selected mobile paths enables saving the routing cost for relaying with the number of stationary hops. As shown in Figure 8, we demonstrate that our algorithm achieves higher utilization of mobile nodes in the entire routing as a more relaxed packet deadline is permitted. We also validate that as the interarrival time of mobile nodes decreases, a higher chance of utilizing mobile nodes is examined in this evaluation.

**Figure 9:**
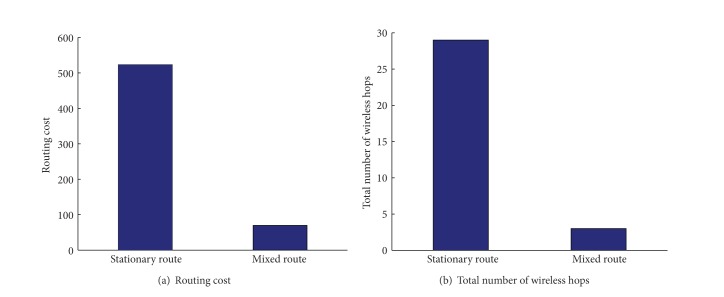
Network performance comparison for the shortest stationary routing versus proposed mixed routing (with the packet deadline of 250 s and the interarrival time of 5 s).

Finally, we compare our proposed routing consisting of both mobile and stationary relays with the shortest-path routing consisting of only stationary relays in Figure 9. Given some delay tolerance and wireless mobile nodes along regular fixed paths, our proposed scheme optimizes the routing path in terms of energy efficiency, significantly reducing routing cost with a factor of 7 (Figure 9(a)). Also, the measured total number of wireless hops from source to destination is greatly decreased with a factor of 9, making packet delivery less sensitive to severe wireless dynamics (Figure 9(b)).

In sum, our proposed routing scheme significantly reduces routing cost and improves packet delivery performance while fully exploiting the allowed packet delivery time within a given deadline, as opposed to the shortest-path routing scheme based only on stationary relay nodes. Also, we find an interesting tradeoff between energy consumption (i.e., routing cost) and packet deadline in the data transportation problem.

## 5. Conclusion

Our novel contribution of this work is the introduction of the* data transportation* problem in ad hoc networks consisting of stationary nodes as well as mobile nodes serving as relays and the development of an efficient distributed routing algorithm. Our proposed algorithm finds energy-efficient routes, balancing stationary paths and mobile paths according to a given hard deadline, in a distributed* greedy* manner. Instead of traversing over only stationary nodes, mobile nodes can effectively be used to carry data packets toward destination as long as the resulting path can meet the given deadline constraint.

Our simulation results indicate that our routing algorithm significantly reduces the total routing cost for delivering data packets from stationary source to stationary destination. With the help of mobile relays, the total number of hop transmissions decreases, and thus packet delivery ratio is greatly improved while guaranteeing packet delivery within the promised packet deadline.

In this work, we solve the problem of data transportation in case of mobile nodes with the predefined regular paths. As a next step, we will more generalize the problem and extend it to the case of mobile nodes with nonregular paths.

Our work is based on a* local* greedy optimization conducted by each stationary node. Interesting future directions could be to formulate this data transportation problem as integer programs and aim to obtain a globally optimal solution.

## Figures and Tables

**Algorithm 1:**
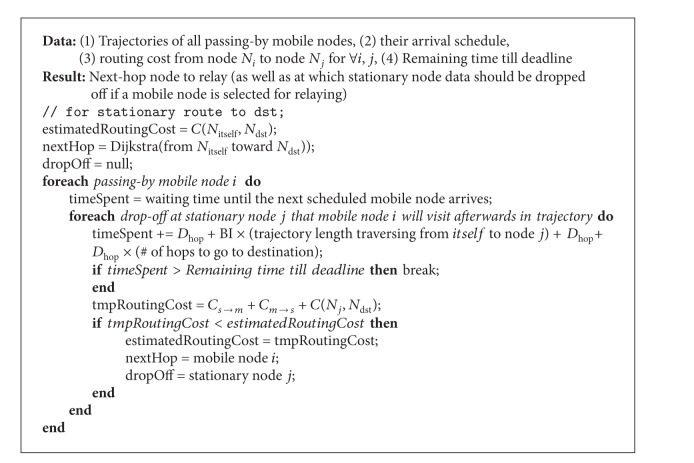
Greedy data transportation algorithm.
